# Repeatability of deuterium metabolic imaging of healthy volunteers at 3 T

**DOI:** 10.1186/s41747-024-00426-4

**Published:** 2024-03-13

**Authors:** Nikolaj Bøgh, Michael Vaeggemose, Rolf F. Schulte, Esben S S Hansen, Christoffer Laustsen

**Affiliations:** 1 The MR Research Centre, Dept. Of Clinical Medicine, Aarhus University, Palle Juul-Jensens Boulevard 99, Aarhus, Denmark; 2 A&E, Gødstrup Hospital, Herning, Denmark; 3 GE HealthCare, Brondby, Denmark; 4 GE HealthCare, Munich, Germany

**Keywords:** Biomarkers (tumor), Brain, Deuterium, Glucose, Magnetic resonance imaging

## Abstract

**Background:**

Magnetic resonance (MR) imaging of deuterated glucose, termed deuterium metabolic imaging (DMI), is emerging as a biomarker of pathway-specific glucose metabolism in tumors. DMI is being studied as a useful marker of treatment response in a scan-rescan scenario. This study aims to evaluate the repeatability of brain DMI.

**Methods:**

A repeatability study was performed in healthy volunteers from December 2022 to March 2023. The participants consumed 75 g of [6,6′­^2^H_2_]glucose. The delivery of ^2^H-glucose to the brain and its conversion to ^2^H-glutamine + glutamate, ^2^H-lactate, and ^2^H-water DMI was imaged at baseline and at 30, 70, and 120 min. DMI was performed using MR spectroscopic imaging on a 3-T system equipped with a ^1^H/^2^H-tuned head coil. Coefficients of variation (CoV) were computed for estimation of repeatability and between-subject variability. In a set of exploratory analyses, the variability effects of region, processing, and normalization were estimated.

**Results:**

Six male participants were recruited, aged 34 ± 6.5 years (mean ± standard deviation). There was 42 ± 2.7 days between sessions. Whole-brain levels of glutamine + glutamate, lactate, and glucose increased to 3.22 ± 0.4 mM, 1.55 ± 0.3 mM, and 3 ± 0.7 mM, respectively. The best signal-to-noise ratio and repeatability was obtained at the 120-min timepoint. Here, the within-subject whole-brain CoVs were -10% for all metabolites, while the between-subject CoVs were -20%.

**Conclusions:**

DMI of glucose and its downstream metabolites is feasible and repeatable on a clinical 3 T system.

**Trial registration:**

ClinicalTrials.gov, NCT05402566, registered the 25th of May 2022.

**Relevance statement:**

Brain deuterium metabolic imaging of healthy volunteers is repeatable and feasible at clinical field strengths, enabling the study of shifts in tumor metabolism associated with treatment response.

**Key points:**

• Deuterium metabolic imaging is an emerging tumor biomarker with unknown repeatability.

• The repeatability of deuterium metabolic imaging is on par with FDG-PET.

• The study of deuterium metabolic imaging in clinical populations is feasible.

**Graphical Abstract:**

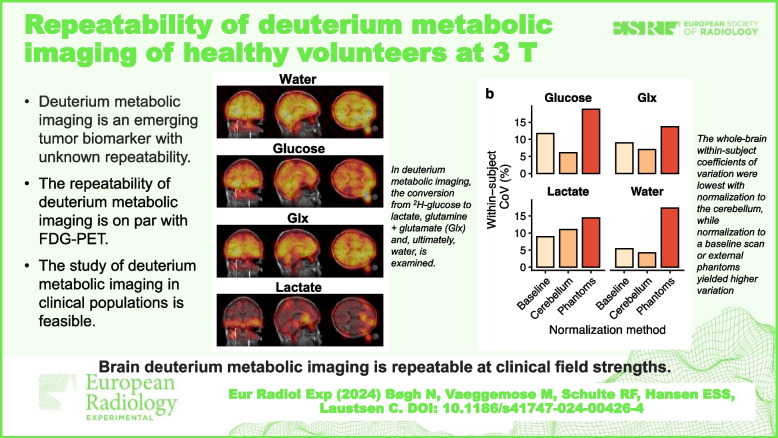

**Supplementary Information:**

The online version contains supplementary material available at 10.1186/s41747-024-00426-4.

## Background

Tumors undergo metabolic reprogramming which promote their growth and spread. Thus, pathway-specific metabolic imaging could guide and personalize cancer care [1]. Emerging magnetic resonance (MR) technologies may come to meet this need by enabling imaging of metabolic probes and their metabolites through the spectral dimension inherent to MR [2, 3]. Recently, deuterium metabolic imaging (DMI) was suggested as a clinically feasible tool for imaging of tumor metabolism [4].

DMI allows imaging of peroral ^2^H-glucose and its conversion into glutamine + glutamate (^2^H-Glx) or ^2^H-lactate. The Glx pool represents citric acid cycle flux, while lactate represents anaerobic metabolism. Thus, DMI can be used to evaluate cancerous reprogramming of two essential pathways. Experimental work shows that scan-rescan DMI is a prompt marker of therapeutic response [5–8]. DMI is viable for clinical trials; however, the initial studies were performed on ultra-high field systems [4, 9–11]. Recently, DMI was shown feasible on clinical field strengths, greatly increasing its availability [12].

This study primarily sought to inform future scan-rescan trials by investigating DMI repeatability at 3 T. To this end, we performed a test–retest DMI study in healthy volunteers. We hypothesized that DMI is as repeatable as tumors ^18^F-fluorodeoxyglucose-positron emission tomography (FDG-PET) [13], corresponding to -10% within-subject coefficients of variation (CoV). Secondarily, we explored the effects of anatomical region, postprocessing, and means of quantification on variability to inform simplified DMI protocols that are easier to implemented in a clinical workflow.

## Methods

### Study design

The study was approved by the Ethics Committee of Central Denmark (1–10-72–78-22) and preregistered at ClinicalTrials.gov (NCT05402566). Healthy volunteers were recruited and gave informed consent from December 2022 to March 2023. They were not allowed to have diabetes or a history of neurological illness. M.V. and R.F.S. are employees of GE Healthcare. The non-industry authors were in complete control of the data and information presented in the study.

All participants were imaged twice. Each session composed of a baseline scan and blood sample, consumption of 75 g [6,6′­^2^H_2_]glucose in 200 mL of water (Cambridge Isotope Laboratories, Tewksbury, USA), and repeated DMI (30, 75, and 120 min) and blood sampling (15, 60, 105, and 150 min). This glucose dose corresponds to a standard oral glucose tolerance test. Blood glucose was determined using point-of-care equipment.

### Deuterium metabolic imaging

MR imaging and DMI were performed using a clinical 3-T system with a modified gradient noise filter (MR 750, GE Healthcare, Chicago, USA) and a 27-cm dual-tuned (^1^H/^2^H) quadrature Tx/Rx volume coil (PulseTeq, Chobham, UK). The anatomical imaging consisted of a 3D T1-weighted inversion-recovery prepped fast spoiled gradient echo sequence (2 × 2 × 2 mm^3^ resolution, repetition time 5.5 ms, echo time 1.7 ms, flip angle 12°, inversion time 450 ms, number of excitations 2). Second-order shimming was employed, and a B_0_ map (IDEAL IQ, 2 × 2 × 2 mm^3^, repetition time 6.9 ms, echo train length 3, echo time 1–5.2 ms) was acquired to ensure its quality.

The deuterium frequency and transmit power were calibrated using the Bloch-Siegert approach on baseline deuterated water [14]. DMI was acquired using three-dimensional chemical shift imaging. The excitation was a soft pulse (flip angle 70°, repetition time 155.8 ms, number of excitations 1678). The readout was a density-weighted spiral (matrix size 10 × 10 × 10, field of view 24 × 24 × 24 cm^3^, spectral points 700, bandwidth 5000 Hz). With four number of excitations, the total scan time was 17:25 min:s. External phantoms with 6.4 mM and 19.15 mM deuterated water in 1.5% agar were included for reference. The coil B_1_^+^-profile was estimated using the double-angle method (flip angle 60/120°, repetition time 1.5 s) on a saline phantom (28 cm, 68 mmol NaCl with 0.2 mmol gadolinium-based contrast).

### Data processing

Data were processed with two parallel pipelines to assess repeatability effects of post-processing. In the simple pipeline, the data were fast Fourier transformed without line broadening, zero filled twice in the spectral dimension, and fitted using a *MATLAB*-implementation of AMARES [15, 16]. In the extended pipeline (Supplemental Fig. S1), the data were denoised using a principal component analyses with automatic rank selection [17, 18], partial-volume corrected using an iterative Lucy-Richardson method [19], and bias field corrected [20]. Fitting was only performed in voxels where the pre-processing signal-to-noise ratio of water was > 5. Voxels with water linewidth > 30 Hz were discarded. Loss of the ^2^H-label and partial radiofrequency saturation were corrected following the procedure of De Feyter et al. [4]. The resulting maps of deuterated water, glucose, lactate, and Glx were Fourier interpolated to the resolution of the anatomical images.

### Image analysis

The metabolite maps were co-registered and normalized to MNI152 space using the *fsl_anat* and *flirt* commands of FSL [21]. Mean metabolite values were calculated for the whole brain, cerebral lobes, cerebellum, caudate nucleus, putamen, and thalamus based on the MNI probabilistic atlas. Apparent metabolite concentrations were estimated from the baseline signal [4]. This was compared to quantification against external phantoms and to internal normalization to the cerebellum.

### Statistics

The primary statistical analysis was calculation of between- and within-subject CoVs estimated by the approach of Lodge et al. (Eq. 1), allowing comparison to their summary of the FDG-PET literature [13].

1 $$between-subject\, CoV = \frac{SD}{mean}$$

2 $$within-subject\, CoV = \frac{SD({Exam}_{2}-{Exam}_{1})}{\sqrt{2}}$$

3 $$repeatability\, coefficient=1.96\times \sqrt{2}\times within-subject\, CoV$$

The CoVs were calculated as relative measures to allow comparison between timepoints with different metabolite concentrations. Effects of time and region of interest were estimated using linear mixed-effect models. The analyses were performed with R version 4.2.3 (R Foundation for Statistical Computing, Vienna, Austria).

## Results

### Participants and metadata

Six male healthy volunteers (age 34 ± 6.5 years [mean ± standard deviation], height 181 ± 6.4 cm, weight 79.6 ± 8.7 kg) were scanned twice 42 ± 2.7 days apart. The baseline fasting blood glucose was 4.7 ± 0.4 mM. It peaked at 6.3 ± 1.7 mM at 60 min after ^2^H-glucose ingestion (Supplemental Fig. S2).

The B_1_^+^-profile of the coil varied ± 7% (Supplemental Fig. S3). DMI quality parameters are presented in Table 1 and raw images are shown in Supplemental Fig. S4. Per examination, 0.2 (range 0 − 5) voxels were excluded, yielding all 48 consecutive examinations of sufficient quality to be included in the analysis.

**Table 1:** Quality control metrics of deuterium metabolic imaging over time calculated from the three central slices of the brain

	Signal-to-noise ratio	Linewidth (Hz)	Cramér Rao lower bounds (%)
Time (min)	Water		
0	10.3 ± 7	9.1 ± 0.9	6.7 ± 2.1
30	12.8 ± 3.9	9.9 ± 0.6	3.4 ± 0.6
75	11.6 ± 2.6	10.2 ± 0.9	3.7 ± 0.9
120	14.2 ± 4.2	10.6 ± 0.6	3.3 ± 0.7
	Glucose		
0	–	–	–
30	4.7 ± 1.5	10.1 ± 0.2	10.7 ± 2.7
75	5.9 ± 2.2	10.1 ± 0.4	8.6 ± 2.9
120	6.1 ± 3	10 ± 0.1	8.5 ± 2
	Glutamate + glutamine		
0	–	–	–
30	1.7 ± 0.5	17.8 ± 2.6	173 ± 48.4
75	3.2 ± 0.7	14.5 ± 1.7	43 ± 21.7
120	4.9 ± 1.5	15.2 ± 1.4	44.6 ± 17
	Lactate		
0	–	–	–
30	1.8 ± 0.6	29.2 ± 6.5	173 ± 55
75	2.3 ± 0.6	26.7 ± 3.4	63.3 ± 26.2
120	2.8 ± 1	26.3 ± 4.6	85.6 ± 42.5

### Metabolite changes and repeatability over time

**Fig. 1:**
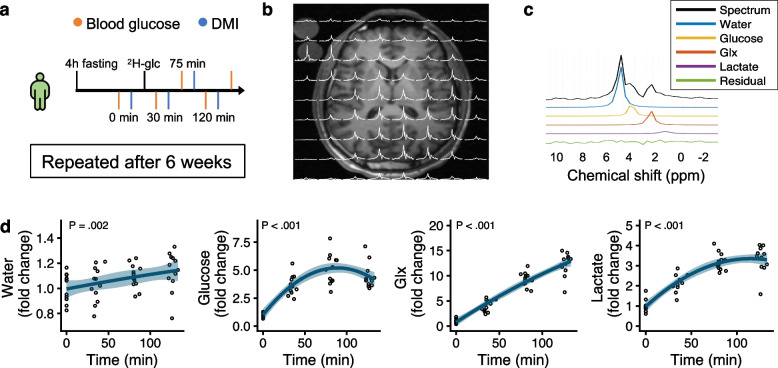
Repeatability of deuterium metabolic imaging (DMI) was investigated in six healthy volunteers. Uptake of glucose and its conversion into lactate, water, and glutamine + glutamate (Glx) was examined after oral intake of 75 g of [6,6-^2^H_2_]-glucose. The imaging was repeated four times for two sessions 6 weeks apart (**a**). Each examination consisted of T1-weighted imaging and ^2^H chemical shift imaging resulting in a 10 × 10 × 10 grid of spectra (**b**). Each spectrum was fitted to water, glucose, lactate, and Glx peaks. Here, a single spectrum from the cortex is shown (**c**). All peaks increased in amplitude over time (**d**); the solid line is a second-degree polynomial fit on the individual data points from the two sessions pooled. The glucose and lactate peaks reached plateau between 75 and 120 min, while the water and Glx peaks continued to increase close to linearly

The metabolite dynamics over time are shown in Fig. 1. When quantified from the baseline signal [4], the apparent whole-brain concentrations of Glx, lactate, and glucose peaked at 3.22 ± 0.4 mM (mean ± standard deviation), 1.55 ± 0.3 mM, and 3.00 ± 0.7 mM, respectively.

Repeatability over time was evaluated to determine the optimal imaging timepoint (Table 2 and Supplemental Figs. S5 and S6). Generally, the within-subject CoV between the two sessions decreased from the 30-min timepoint to the 75- and 120-min timepoints. The variation between individuals decreased with time for Glx, while it stayed constant for the remaining metabolites. Due to better signal-to-noise and repeatability, the 120-min timepoint was used for the subsequent, explorative analyses.

**Table 2:** Whole-brain repeatability and between-subject coefficient of variation (CoV) of deuterium metabolic imaging at 30, 75, and 120 min after ^2^H-glucose administration

	Within-subject CoV (%)	Repeatability coefficient (%)	Between-subject CoV (%)
Time (min)	Water		
30	8.2	22.7	13
75	6.9	19.1	9
120	5.4	15	12.2
	Glucose		
30	11.2	31.3	19.1
75	15.7	43.5	23.7
120	11.7	32.5	19.3
	Glutamate + glutamine		
30	15.6	43.3	25.7
75	8.7	24.3	12.7
120	8.9	24.7	17.7
	Lactate		
30	13.5	37.5	19.9
75	8.5	23.6	16.4
120	8.9	24.7	20.7

*CoV* Coefficient of variation

### Regional variability

**Fig. 2:**
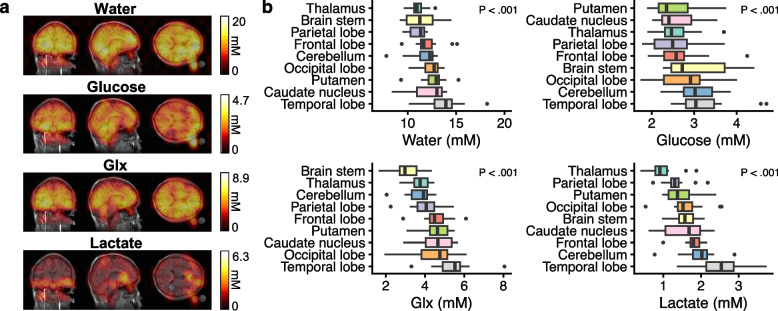
In deuterium metabolic imaging, the water signal is evenly distributed, while metabolite signals display some variation across the brain. The signal outside the brain is from the external phantoms (**a**, a single individual at 120 min is shown, see supplement for grayscale images). Quantification revealed significant differences between regions of the brain that were different between metabolites (**b**). The *p*-values represent a linear-mixed effects model for the effect of region

Regional variation was evaluated to guide focal investigations (Fig. 2). Water and glucose varied -20% across the brain; Glx and lactate varied 42% and 60%. Regional repeatability and variability are presented in Table 3. The within-subject CoV varied from -8 to 10% (frontal and parietal cortices) to -15% (putamen and caudate).

**Table 3:** Within- and between-subject coefficient of variation of deuterium metabolic imaging in different regions of the brain

	Temporal lobe	Occipital lobe	Frontal lobe	Parietal lobe	Cerebellum	Thalamus	Caudate	Putamen	Brain stem
Resonance	Within-subject CoV (%)								
Water	6.3	10.3	4.2	6.2	7.6	4.3	12	6.6	8.7
Glucose	8.9	12.3	15	11.3	10.6	10.8	11.9	15.1	11.9
Glx	11.4	13.2	9.2	8.2	11.3	11.2	16.5	12.3	14.4
Lactate	10.5	13	9.9	7.5	14.9	14.8	19.9	23.6	11.1
	Between-subject CoV (%)								
Water	16	18.2	13	14	13.6	8.5	14.7	11.5	16.6
Glucose	22.1	24.5	23.9	21.6	18.5	20	18.4	23.8	24.6
Glx	21.3	24.8	18.1	20.5	19.1	14.5	21	17	23.6
Lactate	24.7	33.7	16.4	28.8	26.8	41.2	37.7	28.6	22.3

*Glx* Glutamate + glutamine, *CoV* Coefficient of variation

### Variability effects of postprocessing and quantification method

**Fig. 3:**
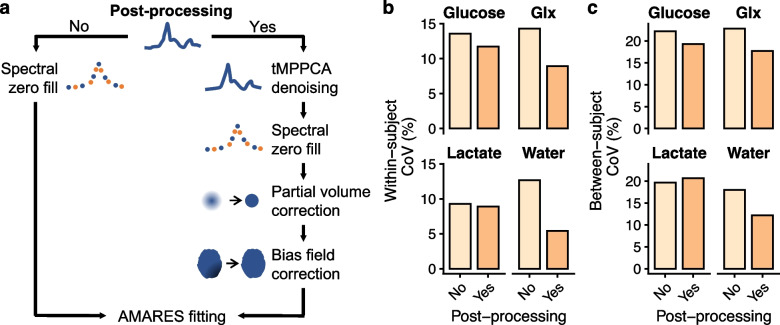
Repeatability effects of post-processing. The deuterium metabolic imaging data were processed using commonly employed techniques (**a**). Coefficients of variation (CoV) within the same individual and between individuals seems to improve with post-processing (**b**). *AMARES*, Advanced method for accurate, robust, and efficient spectral fitting; *Glx*, Glutamate + glutamine; *tMPPCA*, Tensor Marchenko-Pastur principal component analysis

**Fig. 4:**
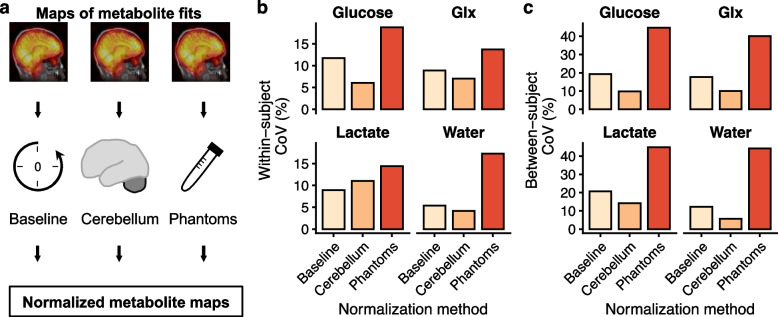
Repeatability effects of normalization. Deuterium metabolic imaging was normalized to either a baseline examination acquired before ^2^H-glucose administration, to the cerebellum in the same examination, or to external phantoms (**a**). Normalization to the baseline allows absolute quantification of apparent metabolite concentrations but requires two examinations. In principle, normalization to external phantoms does the same in a single examination. Internal normalization to the cerebellum is does not give metabolite concentrations. The whole-brain within- and between-subject coefficients of variation (CoV) were lowest with internal normalization to the cerebellum, while normalization to a baseline scan or external phantoms yielded higher variation (**b**,** c**). *Glx* Glutamate + glutamine

When omitting extended postprocessing (Fig. 3), the CoVs increased for Glx and water. Normalization to external phantoms gave metabolite concentrations similar to baseline normalization (2.8 ± 1.4 mM [mean ± standard deviation], 3.2 ± 2.2 mM, and 4.0 ± 2.0 for glucose, Glx, and lactate, respectively). However, this approach yielded large CoVs. Normalization to the cerebellum gave the lowest variation within and between individuals (Fig. 4 and Table 4).

**Table 4:** Whole-brain within- and between-subject coefficients of variation of normalization to a baseline examination, internally to the cerebellum, or externally to phantoms

	Within-subject CoV (%)			Between-subject CoV (%)		
	Normalization			Normalization		
Resonance	Baseline	Cerebellum	Phantoms	Baseline	Cerebellum	Phantoms
Water	5.4	4.2	17.3	12.2	5.7	44.2
Glucose	11.7	6.1	18.8	19.3	9.8	44.7
Glx	8.9	7	13.7	17.7	10	40.1
Lactate	8.9	11	14.4	20.7	14.2	44.9

*Glx* Glutamate + glutamine, *CoV* Coefficient of variation

## Discussion

This study shows that DMI is repeatable with within-subject CoVs below 10% at 120 min after [6,6′­^2^H_2_]glucose ingestion, confirming our primary hypothesis. The variability between subjects was below 20%.

DMI is a new technology, and the optimal imaging protocol is yet to be defined. We found that the signal-to-noise ratio of water and Glx continued to increase until at least 120 min. Opposed to this, the signal-to-noise ratio of lactate and glucose plateaued between 75 and 120 min. Thus, timings need to be considered in study design and interpretation. Generally, the repeatability increased with time. A recent study found good repeatability of indirect deuterium detection using specialized proton spectroscopy at 60 min after administration [22, 23]. Our data suggest that waiting two hours might be optimal for studies where reliable detection of all metabolites is warranted at clinical field strengths.

We performed an explorative analysis of regional variability. Despite varying metabolite concentrations across the brain, the variation between individuals was not decisively larger in regions that are classically considered hard to assess with spectroscopy, suggesting that focal disease can be imaged with DMI regardless of location. We found that normalizing to the cerebellum led to less variation than to external phantoms, likely due to the relatively small size of the phantoms, field inhomogeneities, and shimming isolated to the brain. Internal normalization has the advantage of just requiring a single examination contrary to baseline normalization. Internal normalization is valid if the reference tissue is unaffected by disease, often considered true for the cerebellum in FDG-PET. Thus, we suggest using internal normalization unless absolute metabolite quantification is needed.

Like FDG-PET, DMI images glucose. But there are potentially important differences between the two. First, routine PET uses intravenous trace doses, and care is taken to ensure that the muscles, the liver, and specific cortical areas does not steal the glucose. DMI uses a large dose of oral glucose. This affects the underlying physiology and resembles a tolerance test of the glucose homeostasis where the resting brain is presented for more glucose than it has capacity to utilize [24]. Therefore, we speculate that measures to control peripheral glucose uptake and insulin sensitivity are less important for DMI than in FDG-PET. Additionally, DMI allows imaging of the downstream metabolites of glucose, giving information on specific metabolic pathways in addition to uptake. Here, lactate may be of special interest, as it is now considered not only an anaerobic product but also a modulator of plasticity, memory consolidation, and excitability, among others [25]. Future studies should elucidate how FDG-PET and DMI complements and compares considering the spectral dimension and dose differences between.

Some limitations of clinical DMI should be addressed. The acquired spatial resolution at 3 T is -6 times coarser than modern FDG-PET, and evaluation of smaller brain areas is prone to partial volume effects. Furthermore, the acquisition as performed here is long and thus sensitive to motion. Considerable improvement can be expected through specialized acquisition schemes [26, 27] and at ultra-high field strengths. The present study raises questions for future investigations. First, as also reported by Ruhm et al. [9], the Glx signal seems to increase linearly up to 120 min. As this metabolite is of special interest, the dynamics of Glx should be studied after the 2-h timepoint. In addition, we find dynamics in the lactate resonance, suggesting actual underlying metabolic activity not attributable to lipid contamination. This is in contrast to previous reports [9, 12], and future studies should seek to confirm this finding. Lastly, the sample size of the present study is rather small, and the variation estimates should be interpreted with care. Likewise, we studied the healthy brain, and tumors may behave differently in terms of repeatability and optimal imaging time points.

Our aim was to provide guidance for the design of clinical investigations of DMI. We show that a within-individual change in Glx or lactate larger than ~25% is unlikely to occur be chance. Much larger effects have been demonstrated in experimental work [5–8], suggesting that test–retest clinical trials in brain tumors are viable. Likewise, we show that variation between individuals is small compared to the differences between tumor and healthy brain observed preclinically and clinically [4, 5, 8], suggesting the feasibility of cross-sectional investigations. Lastly, we find that variation is further reduced by internal normalization to a reference tissue, offering a way to simplify DMI compared to the baseline normalization [4, 10, 22, 23].

Collectively, this report shows that DMI is repeatable and feasible at 3 T, enabling sizeable trials of metabolism in a range of brain diseases.

### Supplementary Information

**Additional file 1: Figure S1.** Illustration of the DMI processing pipeline. The spectrum is a single voxel located in the cortex of the middle of the brain. Partial volume correction was performed using an iterative Lucy-Richardson method. The bias field was estimated and corrected for with multiplicative intrinsic component. **Figure S2.** Blood glucose over time after intake of [6,6-2H2]glucose (a) and the correlation between the blood glucose at 60 minutes (the peak) and the DMI whole-brain glucose signal at 120 minutes (b). **Figure S3.** Transmit profile of the employed 1H/2H coil. The transmit field on deuterium was measured across a saline phantom using a double-angle experiment (a). The field varied with ± 7% (b). **Figure S4.** Montage of raw DMI data from a single volunteer at 120 minutes after oral [6,6-2H2]glucose. The root-mean square of the 2H-spectrum is presented, the data were zero filled twice in all directions. **Figure S5.** Bland-Altman plots of repeatability of DMI at 30, 75, and 120 minutes after oral ingestion of [6,6-2H2]glucose. Glx = glutamine+glutamate. **Figure S6.** Correlation plots of repeatability of DMI at 30, 75, and 120 minutes after oral ingestion of [6,6-2H2]glucose. Glx = glutamine+glutamate.

## Data Availability

Data are available at: 10.5281/zenodo.8059573.

## Abbreviations

CoV: Coefficient of variation
DMI: Deuterium metabolic imaging
FDG-PET: ^18^F-fluorodeoxyglucose-positron emission tomography
Glx: Glutamine + glutamate
MR: Magnetic resonance
