# Supplementary material for: Repeatability of deuterium metabolic imaging of healthy volunteers at 3 T
Source: Eur Radiol Exp. 2024 Mar 13;8:44. doi: 10.1186/s41747-024-00426-4 (PMC10933246; doi:10.1186/s41747-024-00426-4)
Supplement: Supplementary file 1 — Additional file 1: Figure S1. Illustration of the DMI processing pipeline. The spectrum is a single voxel located in the cortex of the middle of the brain. Partial volume correction was performed using an iterative Lucy-Richardson method. The bias field was estimated and corrected for with multiplicative intrinsic component. Figure S2. Blood glucose over time after intake of [6,6-2H2]glucose (a) and the correlation between the blood glucose at 60 minutes (the peak) and the DMI whole-brain glucose signal at 120 minutes (b). Figure S3. Transmit profile of the employed 1H/2H coil. The transmit field on deuterium was measured across a saline phantom using a double-angle experiment (a). The field varied with ± 7% (b). Figure S4. Montage of raw DMI data from a single volunteer at 120 minutes after oral [6,6-2H2]glucose. The root-mean square of the 2H-spectrum is presented, the data were zero filled twice in all directions. Figure S5. Bland-Altman plots of repeatability of DMI at 30, 75, and 120 minutes after oral ingestion of [6,6-2H2]glucose. Glx = glutamine+glutamate. Figure S6. Correlation plots of repeatability of DMI at 30, 75, and 120 minutes after oral ingestion of [6,6-2H2]glucose. Glx = glutamine+glutamate. [file 41747_2024_426_MOESM1_ESM.docx]

**Repeatability of deuterium metabolic imaging of healthy volunteers at 3 T**

**ELECTRONIC SUPPLEMENTARY MATERIAL**

**
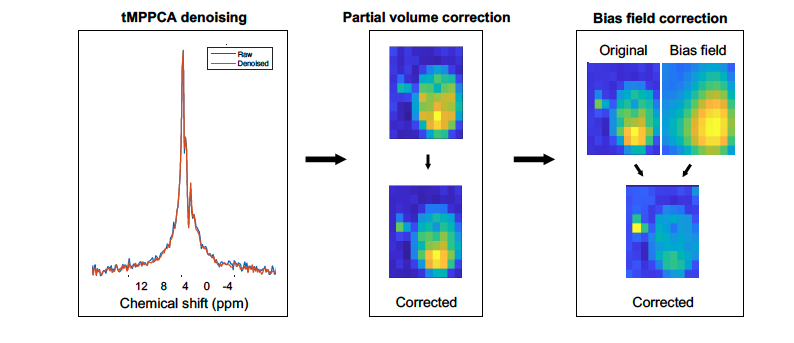
**

**Figure S1:** Illustration of the DMI processing pipeline. The spectrum is a single voxel located in the cortex of

the middle of the brain. Partial volume correction was performed using an iterative Lucy-Richardson

method. The bias field was estimated and corrected for with multiplicative intrinsic component.


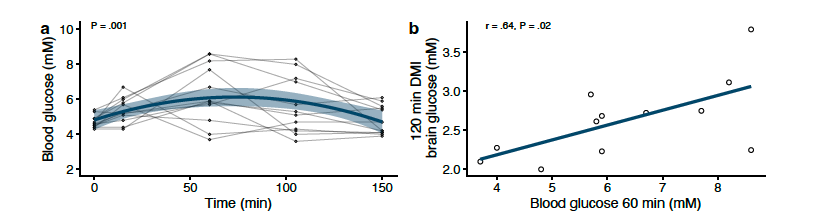


**Figure S2:** Blood glucose over time after intake of [6,6-2H2]glucose (a) and the correlation between the blood glucose at 60 minutes (the peak) and the DMI whole-brain glucose signal at 120 minutes (b).


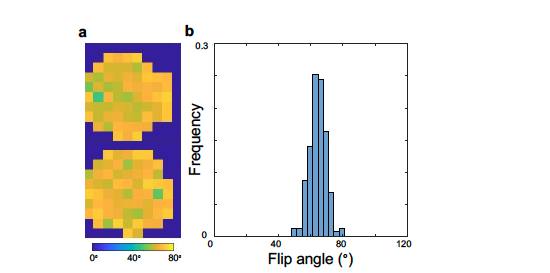


**Figure S3:** Transmit profile of the employed 1H/2H coil. The transmit field on deuterium was measured across a saline phantom using a double-angle experiment (a). The field varied with ± 7 % (b).


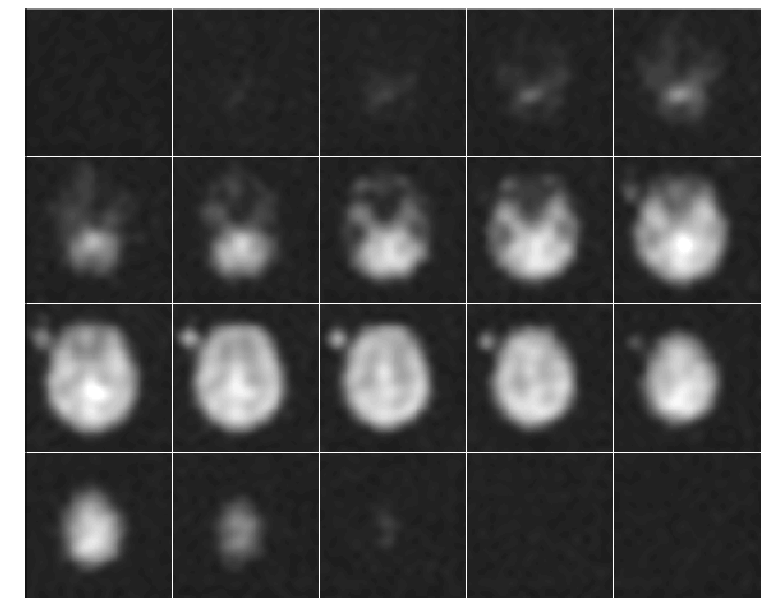


**Figure S4:** Montage of raw DMI data from a single volunteer at 120 minutes after oral [6,6-2H2]glucose. The root-mean square of the 2H-spectrum is presented, the data were zero filled twice in all directions.


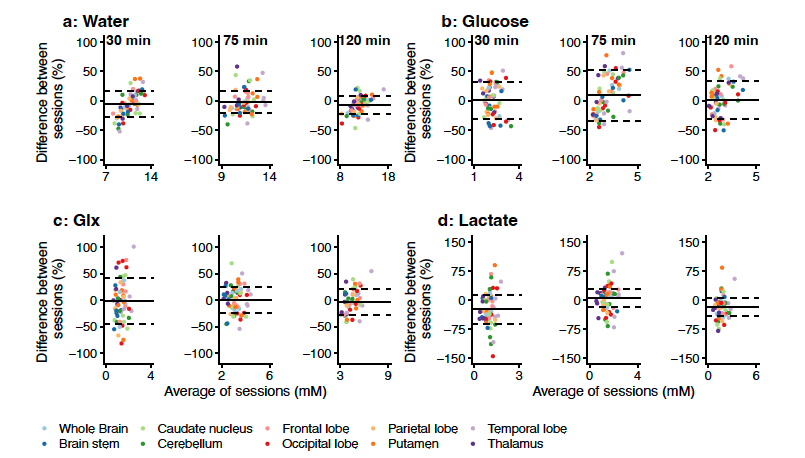


**Figure S5:** Bland-Altman plots of repeatability of DMI at 30, 75, and 120 minutes after oral ingestion of [6,6-2H2]glucose. Glx = glutamine+glutamate


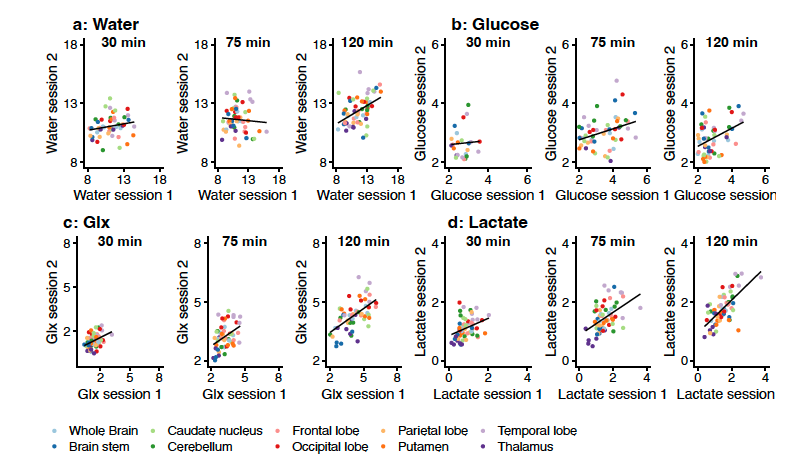
.

**Figure S6:** Correlation plots of repeatability of DMI at 30, 75, and 120 minutes after oral ingestion of [6,6-2H2]glucose. Glx = glutamine+glutamate.
